# Supplementary material for: Left ventricular longitudinal strain variations assessed by speckle-tracking echocardiography after a passive leg raising maneuver in patients with acute circulatory failure to predict fluid responsiveness: A prospective, observational study
Source: PLoS One. 2021 Sep 30;16(9):e0257737. doi: 10.1371/journal.pone.0257737 (PMC8483378; doi:10.1371/journal.pone.0257737)
Supplement: Attachment — Submitted filename: Response to Reviewers.docx [file pone.0257737.s001.docx]

*Robert Ehrman*, MD, MS

Academic Editor

PLOS ONE

Marseille, Tuesday the 6^th^ July 2021

Dear editor,

We thank academic editor Robert Ehrman and anonymous referees #1 and #2 for their generous comments and suggestions on the manuscript which helped us to improve the article in many ways. We considered the comments with care and acted on the majority of the points raised by the reviewers. We clarified the points we were asked to and gave details on statistical methods as required.

We were asked to discuss changes in the article’s title, which basically was “***Left ventricular longitudinal strain variations assessed by speckle-tracking echocardiography after a passive leg raising maneuver to predict fluid responsiveness: A prospective, observational, monocentric study”.***

We suggest, as a new title,***” Left ventricular longitudinal strain variations assessed by speckle-tracking echocardiography after a passive leg raising maneuver in patient with acute circulatory failure to predict fluid responsiveness: A prospective, observational, monocentric study”.***	We would rather not change the title to “[…] does no predict fluid responsiveness” as we would like the readers to understand how important it is to evaluate patient’s load conditions before construing left-ventricular longitudinal strain values, although LV-LS variations are definitely not a valuable tool to assess preload dependency as shown is this study. We, of course, are disposed to discuss the title if required.

Please find below our answers to all referees’ comments, presented in the same order as in the review. A revised version of the manuscript was submitted to address their concerns.

Thank you for considering our manuscript for publication as an original article in PLOS ONE.

Sincerely,

Clemence ROY

| **Comment from Academic Editor** | **Answer** | **Amendments** |
| --- | --- | --- |
| Can you provide more detail about the repeated measures regression model? It seems like LV-LS was the outcome and PLR + vs - was the predictor--is this correct?  While many of the characteristics were similar in the PLR groups, I wonder if there are any potential confounders that could be adjusted for in the model? there is probably not a "right" answer, and an overfit model is certainly not advantageous to anyone, but I think that more detail about the model itself and the decisions and assumptions that went into to would provide clarity and strength to the paper | Thank you for your remark. In the presented regression model for repeated measures, preload dependency status (represented by PLD+ or PLD-) was the predictor, and the myocardial strain response to PLR, evaluated by LV-LS, was the outcome.  In this model, LV-LS is the dependent variable, that varies between two time points T0 and T1. PLD+ and PLD- are independent of T0 and T1.  The intra-subject effect shows an increase of LV-LS absolute value, in both groups (PLD+ and PLD-) between T0 and T1 (with *p = 0.001* in the regression model). But there is no inter-subject statistical difference, with *p = 0.13* when we compared the two groups.  We conducted a univariate analysis which found no association between the major confounding factors (e.g aetiology of ACF, norepinephrine infusion, lactate level, sedation). The correlation matrix showed no evidence for correlation, apart for expected couples (i.e septic shock/norepinephrine infusion).  We conducted further statistical analysis as you suggested, adjusting the regression model on two variables which were close to significance in the univariate analysis with p<0.2 : septic shock and norepinephrine infusion.  When adjusting the regression model for repeated measures on septic shock we found *p = 0.292*, when adjusting on norepinephrine infusion we find *p = 0.612*.  We discussed this important point in the revised manuscript. | Page 9, line 207  Page 13, lines 281-283 |
| Another important idea is that of patient-level heterogeneity and its relationship to PLR status and echo parameters. You have alluded to this in the conclusion section, and I certainly agree with the ideas presented therein. however, I think this is a very important part of the paper and would be more appropriately located in the discussion or limitations section, perhaps with a bit more detail/explanation | Thank you for this comment.  Changes were made in the discussion section.  “The studied population in this cohort is representative of patients admitted in ICUs with ACF, with varied pathologies leading to circulatory failure such as sepsis, haemorrhage, heavy surgery followed by systemic inflammatory response. We chose to enroll patients admitted for any cause of ACF, which led to a great heterogenicity in our population, regarding clinical features and the pathophysiological mechanism responsible for a circulatory failure. Our final groups, PLD+ and PLD-, were not akin concerning the aetiology of ACF. As matter of fact, there were significantly more patients admitted for septic shock in the PLD- than in the PLD+ group, which possibly biased our analysis.” [15] | Pages 15-16, lines 332-339 |
| **Comments from Reviewer #1** | **Answer** | **Amendments** |
| The PLR maneuver can cause translation of the heart and affect VTI assessments. Please comment on this in the limitations | Thank you for this remark we for sure agree with.  However, it has been shown in a meta-analysis by Cherpanath *et al*. [15] that the pulse contour derived-cardiac output, as well as the cardiac output measured by esophageal Doppler, transthoracic echocardiography or bioreactance had a similar diagnostic performance to assess cardiac output variations after a passive leg raising maneuver.  Possible translation of the heart during PLR does not seem to have a negative impact on the evaluation of CO surrogates such as LVOT-VTI. Furthermore, in clinical practice, delta VTI after PLR was acknowledged a validated tool with an almost perfect area under the curve (Monnet *et al.* [11]) and is currently consider as a gold standard for fluid responsiveness diagnosis in large studies (Vignon et al, Comparison of Echocardiographic Indices Used to Predict Fluid Responsiveness in Ventilated Patients, AJRCCM 2017). |  |
| The study omitted one third of eligible patients due to technical limitations. This may bias the sample, as these patients might be sicker. Please comment on this in the limitations. | Thank you for this interesting question.  Within the 49 excluded patients, 17 had poor echogenicity. Most of them were admitted after major thoracic surgery which is an important part of our ICU recruitment. Bandage, surgical wounds, leaft us poor echographic window.  However, excluded patients had a similar mean SAPSII score to included patients. Unfortunately, we did not assess SOFA score in the non-included population but severity of the patients was never the reason of their non-inclusion. |  |
| I have concerns for a type I error. E/e’ is a surrogate for preload, or more specifically LVEDP. There is no difference between PLD+ and PLD- patients at T0 for E/e’. E/e’ is a marker of preload, not preload responsiveness, so it’s not completely incorrect if there is no difference between groups. However, E/e’ decreased with the PLR in both PLD+ and PLD- patients, which would suggest inconsistency in E/e’ measurement, in PLD assessment, or both. Please clarify.  Additionally, the main outcome (ΔStrain after PLR) is perhaps not the most obvious variable to select, and it barely achieves significance, while several other hypotheses were assessed. Please confirm in the methods that the delta longitudinal strain after a PLR was the a priori primary assessment, or please correct these tests of significance for multiple hypothesis testing. | Thank you for this comment.  As far as E/e’ is concerned:  We agree that E/e’ variation after PLR can be used to control PLR efficiency. However, E/e’ variation is not very accurate to quantify small transitory variations of preload. One reason might be that an increase of e’ is observed in preload dependent patients after correcting hypovolemia as shown by Mahjoub and colleagues (Mahjoub et al, Improvement of left ventricular relaxation as assessed by tissue Doppler imaging in fluid-responsive critically ill septic patients, Int Care Med, 2012).  As reviewer#1 noticed, E/e’ was more specifically validated as a marker of LV filling pressure, allowing clinicians to anticipate the risk of fluid overload when a volume expansion is considered to treat ACF.  Studies focused on evaluation of preload dependency found no association between E/e’ and fluid responsiveness. For example, Lamia et al ([14], Table3) found a similar E/e’ ratio between responder and non-responder patients, before and after volume expansion.  Thus, we are not surprised to find a non-significant decrease of this parameter, after PLR, in both our groups.  Second part of Rewiewer1 remark:  We confirm our main outcome was ΔLV-Strain after PLR, and the study was designed on the hypothesis that ΔStrain after PLR would be greater in PLD+ group than in PLD-. Our hypothesis was already raised in the paper from Nafati and colleagues [21] showing that LV-GLS value increased after volume expansion in preload dependent patients.  Statistical analysis were based and designed on this only hypothesis. |  |
| Page 4 and 5. VTI after PLR is perhaps the most generalizable and generally the most accurate method of assessing preload responsiveness, but it is misleading to call it the gold standard. Please revise | Thank you for this comment.  Because the area under curve of VTI variations after PLR is almost perfect to predict fluid responsiveness, we chose this marker as a Gold standard to avoid unnecessary fluid loading in preload independent patients. However, according to your comment, we removed this term in the all manuscript. | Page 4, lines 82-83 and 108 |
| Table 1: Sex is misspelled (Sexe) | Noted. | Table 1, page 11 |
| **Comments from Reviewer#2** | **Answer** | **Amendments** |
| Title: It would be better to point out the subjects (patients with ACF) included in this study in the title. Additionally, the title should indicated the conclusion that variations in LV-LS after PLR is not a discriminating criterion to predict fluid responsiveness. Discuss the title in the letter to editor | Revised title now includes details on the studied population.  Please refer to the letter to editor to find more details about discussion around the title. | Title, first page. |
| Methods: fluid responsiveness should be defined in the Methods | We used evaluation of preload dependency to predict fluid responsiveness, but did not assess fluid responsiveness in our cohort as patients did not receive any fluid. In the method section we defined what we considered preload-dependency : a LVOT-VTI variation greater than 10% after a passive leg raising maneuver, a validated method. | Page 7, lines 145-147 |
| Results : (page13, line271): This study compared the variation of LV-LS in whole subjets after PLR. The variations of other parameters after PLR such as LV-SSR, RV-LS and LA-LS which were assessed by speckle tracking analysis should be added in this analysis to test their prediction value for fluid responsiveness | These data are now presented in the results section. | Page 14 , lines 296-307 |
| Results(page13, line270-274): Significant differences of mean LV-LS variations between T0 and T1 was observed in the whole population (n=60, p=0.04), but not in the PLD+ group (n=33, p=0.04) and PLD- group(n=27, p=0.25). Additionally, these variations of LV-LS after PLR were not statistically different between the groups (p=0.08)? The results might result from the small numbers of subjects, and statistical power test should be added in this study. | We calculated the number of required subjects according to our primary goal which was to determine the sensitivity and specificity of LV-LS variations to assess preload dependency. We assumed, in accordance with literature, that 50% of our ACF population would be PLD+ and 50% PLD-, with roughly balanced groups. Taking that into account, for an aera under the curve > 0.85 with a confidence interval of 0.20 (CI 95% 0.75-0.95) and an alpha risk of 5% we found a required number of subjects of 60 with 50% in each group. Required number of subjects could not be calculated for the LV-LS variation, as we had no idea if 1) it would vary , and 2) in what proportion. These variations were never studied before.  The “a posteriori” calculated required number of subjects, that would have been needed to ascertain a difference in LV-LS variation between our groups is 400. We, for sure, must acknowledge a lack of power... We added this important limitation in the discussion section. | Page 16-17, lines 352-363 |
| Is there any liner correlation between variations of LV RVOT VTI and LV-LS after PLR? | Unfortunatelly, no statistical correlation was found between LVOT-VTI variations and LV-LS variations after PLR.  - In the whole population (n=60) the Pearsons’ coefficient was 0.183, *p = 0.161*.  - In the PLD+ group (n=33) the  Pearsons’ coefficient was -0.015, *p = 0.934*  - In the PLD- group (n=27) the  Pearsons’ coefficient was -0.051, *p = 0.800* |  |
| Conclusion(page18, line384-385): This study did not test the correlations between LV-LS and afterload and intrinsic myocardial function. You should add related statistical analysis or modify the conclusion. | Noted, we modified the conclusion | Page 18, lines 405-406 |
| 8.What is the advantages and innovations of this study? | We built this observational study after the results of Nafati and colleagues’ study was published [21].  Indeed, that was the first time a study showed a variation of LV-GLS after a volume expansion in a preload dependent population of patients. We decided to question the problem from the other side and evaluate LV-LS variation after PLR as a predicting marker of preload dependency.  In current practice, LVOT-VTI variation after PRL is already a reliable marker, easy to learn and available at bedside in most situations. From that point of view, evaluation of the LV-LS variation could appear pointless.  But, two points must be discussed here.  First, this study shows that the Speckle tracking technology can be used in ICUs in clinical practice when available, as a simple tool that can precisely evaluate the systolic segmental function of the myocardium, following myocardial strain after severe myocarditis for example.  Cardiologists now use it as a routine evaluation.  Second, our study raises a caution here : interpretation of myocardial strain parameters in ICU patients implies that clinician should assess the haemodynamic status of the patients before analyzing any myocardial strain measurements. |  |
| **Journal requirements** | **Answer** | **Amendmends** |
| PLOS ONE's style requirements | Instructions followed |  |
| What type of consent you obtained | Written consent was obtained | Pages 5-6, lines 115-117 |
| Competing interest section | Completed.  Marc Leone, co-author, added his competing interest in this section as well. | Page 19, lines 416-421 |
