# Supplementary material for: Left ventricular longitudinal strain variations assessed by speckle-tracking echocardiography after a passive leg raising maneuver in patients with acute circulatory failure to predict fluid responsiveness: A prospective, observational study
Source: PLoS One. 2021 Sep 30;16(9):e0257737. doi: 10.1371/journal.pone.0257737 (PMC8483378; doi:10.1371/journal.pone.0257737)
Supplement: Attachment — Submitted filename: Response to Reviewers.docx [file pone.0257737.s002.docx]

*Robert Ehrman*, MD, MS

Academic Editor

PLOS ONE

Marseille, Thursday September 2nd, 2021

Dear editor,

We thank again academic editor Robert Ehrman and anonymous referees #1 and #2 for their second review, with precise comments focused on the presented echocardiographic parameters, how they are linked to the haemodynamic status of the patient and to the aetiolgy of the acute circulatory failure.

We closely considered the comments and tried to clarify the points that were raised in the first and second review, as concisely as possible. Yet, the discussion section might appear a bit long and we are willing to shorten it if required.

Please find below the listed changes made after considering comments from Academic editor and Referee #1. A revised version of the manuscript was submitted to address their concerns.

Thank you for considering our manuscript for publication as an original article in PLOS ONE.

Sincerely,

Clemence ROY

| **Previous comments discussed again in the second reviewing** | **Answer** | **Amendments** |
| --- | --- | --- |
| Reviewer#1  I have concerns for a type I error. E/e’ is a surrogate for preload, or more specifically LVEDP. There is no difference between PLD+ and PLD- patients at T0 for E/e’. E/e’ is a marker of preload, not preload responsiveness, so it’s not completely incorrect if there is no difference between groups. However, E/e’ decreased with the PLR in both PLD+ and PLD- patients, which would suggest inconsistency in E/e’ measurement, in PLD assessment, or both. Please clarify. | Thank you for this comment.  As far as E/e’ is concerned:  We agree that E/e’ variation after PLR can be used to control PLR efficiency. However, E/e’ variation is not very accurate to quantify small transitory variations of preload. One reason might be that an increase of e’ is observed in preload dependent patients after correcting hypovolemia as shown by Mahjoub and colleagues (Mahjoub et al, Improvement of left ventricular relaxation as assessed by tissue Doppler imaging in fluid-responsive critically ill septic patients, Int Care Med, 2012).  As reviewer#1 noticed, E/e’ was more specifically validated as a marker of LV filling pressure, allowing clinicians to anticipate the risk of fluid overload when a volume expansion is considered to treat ACF.  Studies focused on evaluation of preload dependency found no association between E/e’ and fluid responsiveness. For example, Lamia et al ([14], Table3) found a similar E/e’ ratio between responder and non-responder patients, before and after volume expansion.  Thus, we are not surprised to find a non-significant decrease of this parameter, after PLR, in both our groups. |  |
| **Comment from Academic Editor** | **Answer** | **Amendments** |
| Thank you for your revisions. I am in agreement with Reviewer #1 in that the paper would be strengthened by including in the manuscript many of the responses to the reviewer comments that explain the underlying reasoning for many of the decisions that were made. | See Answer to Reviewer#1 |  |
| **Comments from Reviewer #1** | **Answer** | **Amendments** |
| Reviewer #1: I thank the authors for responding to the comments raised by the editor and reviewers. I agree with many of their responses. However, the reason for raising these comments is that I expect other interested readers may ask similar questions. Therefore, while responding to the comments satisfies me, other readers won't have the same information unless they delve into the review history to see this. Please consider some minor revisions to the manuscript to include the important information you provide in your response.  Most of my comments were addressed obliquely with revisions made for the editor or other reviewer--there is mention of possible selection bias, and mention of the accuracy of PLR. However, the discordance between E/e' and PLR is of interest, and the authors respond wonderfully with a succinct description of the physiologic basis for why this could be discordant. I'd like to see that in the discussion. It would strengthen the plausibility of the findings and educate the readers. This merges well with the discussion of patient-level heterogeity. | Thank you for these perceptive remarks that helped us to clarify the discussion. In this new version of the manuscript, we tried to give more details to the readers about how the echocardiographic markers evaluated in ICU patients with acute circulatory failure, should be interpreted with caution.  Indeed, most markers that we use to assess preload-dependency, left ventricular end diastolic pressure, systolic or diastolic ventricular function, should be interpreted within the framework of the clinical history of the patient, and especially the aetiology of the circulatory failure.  Thus, we added a paragraph in the discussion section responding to reviewer#1 sensible remark concerning the relationship between E/e’ and preload, and about the E/e’ variations after a passive leg raising maneuver.  We also insisted, on the importance of the patient-level heterogeneity in the presented study, which is probably partly responsible for the negative results.  Therefore, this paragraph was added in the text:  “Regarding the usual echocardiographic markers that were evaluated at T0 and T1, there was a significant difference concerning cardiac output (and surrogates as LVOT-VTI) between the PLD+ and PLD-, but no significant difference was observed concerning echocardiographic markers of left-ventricular end diastolic pressure (LVEDP). Indeed, there was no difference between mean E, E’ lat, nor E/E’lat between PLD+ and PLD- and no significant variation of these markers between T0 and T1 in the whole population. Yet, some authors showed a relationship between E’lat and preload. Mahjoub *et al*. [45] showed a significant increase of E’lat (and a resultant decrease in E/E’lat) in a population of septic shock patients with diastolic dysfunction (E’Lat < 0.12cm/s) receiving a 500 ml crystalloid administration. These results suggest an improvement of LV relaxation with the correction of hypovolemia in patients with septic shock and diastolic dysfunction. Lamia *et al.* [14] studied the variations of echocardiographic markers of preload and LVEDP using a PLR maneuver, followed by a volume expansion and found similar results as ours, i.e. a non-significant variation of E, E’Lat and E/E’lat between responders and non-responders following PLR or crystalloid infusion. This study also concerned patients with various aetiology responsible for circulatory failure. Thus, if an increase of E’lat can be observed in preload-dependent patients after correcting hypovolemia, E/E’lat variation is not very accurate to quantify small transitory variations of preload and should rather be used as a marker of LVED, to anticipate the risk of fluid overload when a volume expansion is considered to treat ACF.”  We added one reference (number 45, Mahjoub Y, *et al*. Improvement of left ventricular relaxation as assessed by tissue Doppler imaging in fluid-responsive critically ill septic patients. Intensive Care Med. 2012) to document this new paragraph | Lines 376-394  Lines 594-597 |
